# Acetoin production from lignocellulosic biomass hydrolysates with a modular metabolic engineering system in *Bacillus subtilis*

**DOI:** 10.1186/s13068-022-02185-z

**Published:** 2022-08-24

**Authors:** Qiang Wang, Xian Zhang, Kexin Ren, Rumeng Han, Ruiqi Lu, Teng Bao, Xuewei Pan, Taowei Yang, Meijuan Xu, Zhiming Rao

**Affiliations:** 1 Key Laboratory of Industrial Biotechnology of Ministry of Education, School of Biotechnology, Jiangnan University, Wuxi, Jiangsu 214122 People’s Republic of China; 2 Department of Bioengineering, The University of Illinois at Urbana-Champaign, Urbana, IL USA

**Keywords:** Acetoin, Low-cost biomass, *Bacillus subtilis*, Carbon flux, Population cell density-induced promoter

## Abstract

**Background:**

Acetoin (AC) is a vital platform chemical widely used in food, pharmaceutical and chemical industries. With increasing concern over non-renewable resources and environmental issues, using low-cost biomass for acetoin production by microbial fermentation is undoubtedly a promising strategy.

**Results:**

This work reduces the disadvantages of *Bacillus subtilis* during fermentation by regulating genes involved in spore formation and autolysis. Then, optimizing intracellular redox homeostasis through Rex protein mitigated the detrimental effects of NADH produced by the glycolytic metabolic pathway on the process of AC production. Subsequently, multiple pathways that compete with AC production are blocked to optimize carbon flux allocation. Finally, the population cell density-induced promoter was used to enhance the AC synthesis pathway. Fermentation was carried out in a 5-L bioreactor using bagasse lignocellulosic hydrolysate, resulting in a final titer of 64.3 g/L, which was 89.5% of the theoretical yield.

**Conclusions:**

The recombinant strain BSMAY-4-P_*srfA*_ provides an economical and efficient strategy for large-scale industrial production of acetoin.

**Supplementary Information:**

The online version contains supplementary material available at 10.1186/s13068-022-02185-z.

## Background

Acetoin (3-hydroxy-2-butanone) is widely used in food, pharmaceutical and chemical industries [1, 2]. Because of these important applications, in 2004, the US Department of Energy listed it as one of 30 bio-based platform chemicals that should be given higher priority for development and utilization [3]. The acetoin production methods include chemical and biological synthesis [4]. The raw materials for chemical synthesis methods are mainly derived from petroleum resources, such as diacetyl and 2,3-butanediol. Currently, the continuous reduction of petroleum resources and the environmental hazards of chemical production limit the large-scale production of acetoin by chemical synthesis methods. Furthermore, when acetoin is produced by chemical synthesis, the by-products often have certain toxicity, which limits its application in food, medicine and other industries [5–7]. Therefore, increased attention has been devoted to biological synthesis methods, which can be divided into enzymatic conversion and microbial fermentation. The enzymatic conversion approach is unsuitable for industrial acetoin production because the precursor substances are expensive and not readily available, and the production process is complex [8].

In contrast, the microbial fermentation method is less affected by natural environmental conditions, the production process is easy to control, green and environmentally friendly, and is conducive to maintaining the stability of product quality. In addition, microorganisms have an active metabolism and grow and multiply rapidly, allowing the production of a batch of fermented products in a short period. Therefore, fermentation is currently the most competitive approach for acetoin production. Microbial fermentation for acetoin production usually uses glucose as the primary carbon source, which is uneconomical for industrial production [9, 10]. Therefore, it would be more advantageous to use second-generation biofuel feedstocks, such as the hydrolysis products of lignocellulose, for the fermentation production of acetoin [11]. In the future, using low-cost and renewable plant biomass as a raw material for production is undoubtedly a promising production strategy that is more conducive to the industrial production of the product.

In nature, many bacteria can synthesize acetoin, including *Klebsiella* [12], *Bacillus* [13], *Enterobacter* [6], *Lactococcus* [14], and *Serratia* [15]. However, acetoin is generally present as a by-product of 2,3-butanediol and diacetyl in the metabolism of these strains that can synthesize acetoin naturally. Direct fermentation using these microorganisms results in a deficient synthesis of acetoin, making it challenging to meet the demands of industrial production and the pathogenicity of some strains. In order to solve the problem of the low yield of acetoin synthesized by microorganisms, the researchers obtained a batch of high-yielding strains through mutagenesis screening and fermentation optimization [16]. But the genetic stability of these strains has been altered through mutagenesis, and there are too many uncertainties and potential risks in product safety. Therefore, researchers gradually began to perform metabolic engineering of some model strains, aiming to change the carbon flow distribution of the strains through biotechnology and increase acetoin production [17]. *B. subtilis* is a typical industrial strain for food safety. It has been widely used in metabolic engineering due to its non-pathogenicity and lack of significant codon preference [18, 19], which is a very promising chassis cell with obvious advantages for the industrial mass production of acetoin.

**Fig. 1:**
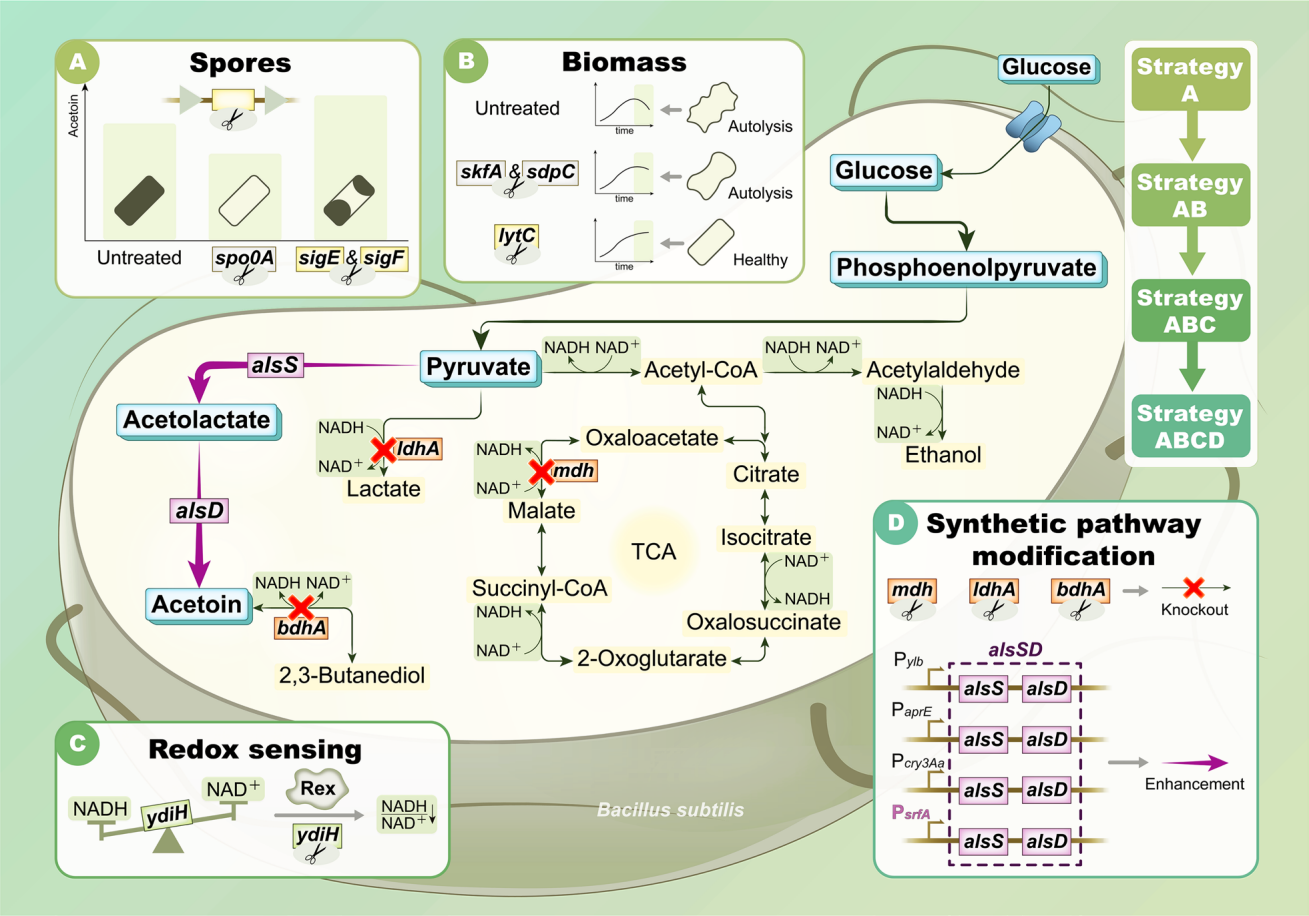
Biotechnological strategies for acetoin production by *B. subtilis*. This work blocked spore formation at different stages and mitigated autolysis in strains by regulating genes for peptidoglycan hydrolase and cell cannibalism. Subsequently, NADH/NAD^+^ was regulated by the redox global regulator Rex to optimize the allocation of carbon flux levels, followed by blocking the main pathway of lactate, succinate, and 2,3-butanediol production. Finally, the population cell density-induced promoter enhances the enhancement of α-acetolactate synthase and α-acetolactate decarboxylase. Bold arrows indicate overexpressed genes in this study, and red crosses indicate disrupted genes

In this study, we first blocked the spore synthesis pathway and knocked out essential genes that control cell autolysis, which improved the efficiency of substrate utilization by the strain. In addition, to address the problem of low acetoin production and high by-products during fermentation [20], we redistributed the carbon flux of the synthesis pathway through the intracellular redox-sensing regulator Rex [21, 22] and explored the relationship between changes in intracellular NADH/NAD^+^ levels and acetoin production during fermentation. The main by-product synthesis pathways competing with acetoin synthesis were combinatorially blocked, and their effects on acetoin production were analyzed. To further improve the efficiency of acetoin production while avoiding biomass reduction in the pre-fermentation period [23], we chose to use a population cell density-induced promoter to enhance the expression of α-acetolactate synthase (ALS, encoded by the gene *alsS*) and α-acetolactate decarboxylase (ALDC, encoded by the gene *alsD*), and increase the efficiency of the catalytic synthesis of acetoin from pyruvate (Fig. 1).

## Results and discussion

### Knockout spore-formation genes to increase AC production by reducing nutrient wastage

Due to the presence of some organic compounds in the bagasse hydrolyzate, these substances accelerate spore formation in *B. subtilis* [24, 25]. The formation of spores consumes a lot of energy and substances, such as enzymes required for spore synthesis, various regulatory factors, lipids and dipicolinic acid [26], reducing the substrate utilization. In this study, we blocked spore formation at the initiation (encoded by *spo0A*) and asymmetric division stages (encoded by *sigE* and *sigF*). And we explored the effect of blocking the spore-formation pathway on acetoin synthesis in a fermentation experiment.

**Fig. 2:**
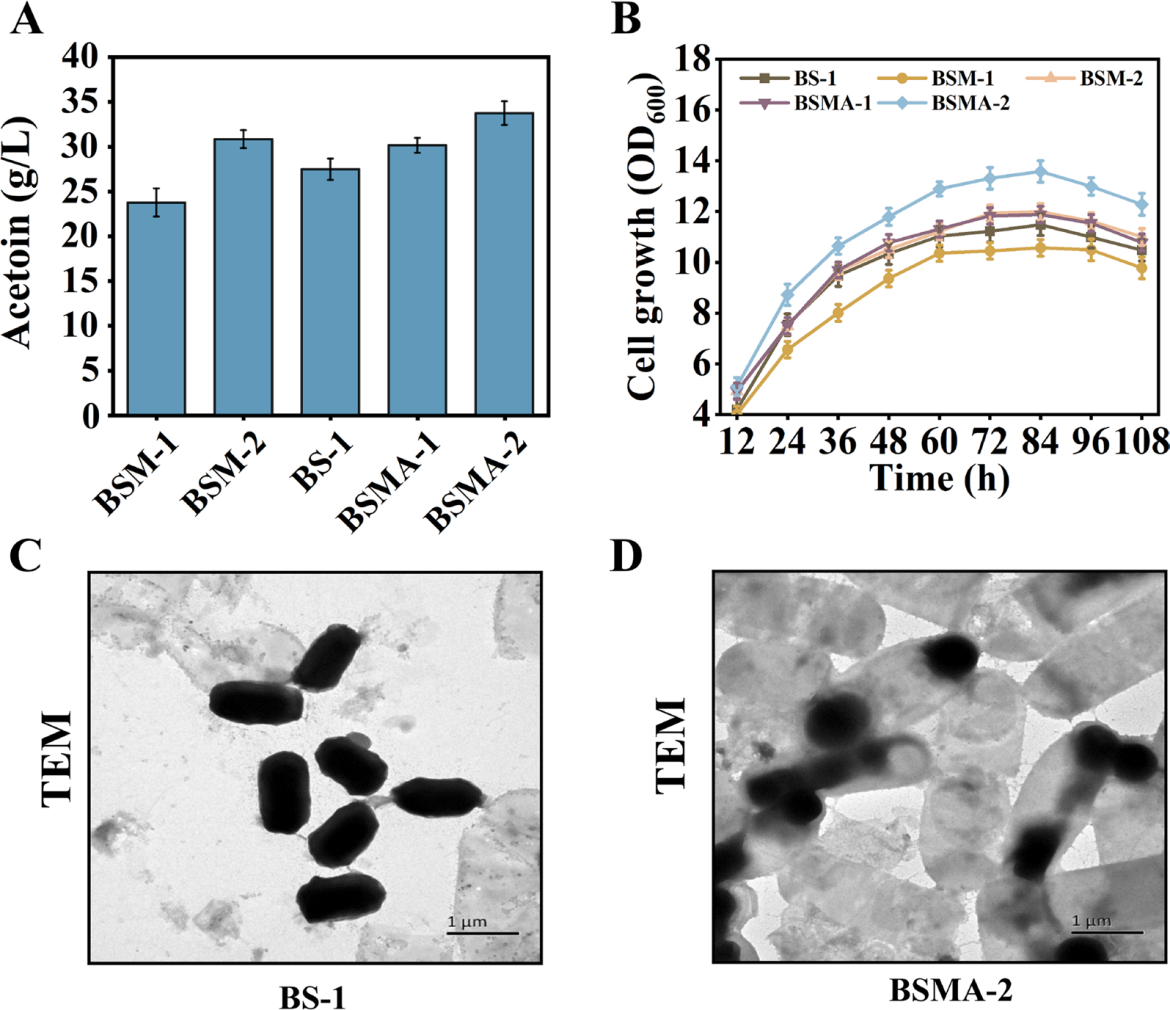
**A** Effect of blocking the formation of spores at different times and knocking out different types of autolysis-related genes on acetoin synthesis. **B** Trends in biomass of different recombinant strains over time. **C** and **D** Transmission electron micrographs (TEM) of the control strain BS-1 and recombinant strain BSMA-1. All assays were performed in triplicate, and the standard deviations of biological replicates are indicated by error bars

The control strain *B. subtilis* 168 (BS-1) and the recombinant strains *B. subtilis* 168Δ*spo0A* (BSM-1) and *B. subtilis* 168Δ*sigE*Δ*sigF* (BSM-2) were used in this study with bagasse hydrolysate as the substrate. The results indicated that the rate of sugar consumption was the same for all three strains, suggesting that the knockout of the *spo0A*, *sigE*, and *sigF* genes did not have a regulatory relationship with the related genes in the glycolytic pathway. Regarding acetoin production, the maximum titer for BSM-1 was only 23.7 g/L,13.8% lower than the 27.5 g/L observed for the control strain BS-1 (Fig. 2A). In contrast, the acetoin production by BSM-2 was improved compared with BS-1, with the titer reaching 30.8 g/L under the same conditions. Meanwhile, the biomass of the recombinant strain BSM-1 was significantly lower than that of the control strain BS-1, whereas there were no apparent variations between the control strain and the BSM-2 (Fig. 2B).

The exact reason for the knockout of the *spo0A* gene inhibiting bacterial growth is still unclear, and Takeko Kodama [27] speculated that the mutant with the gene *spo0A* deleted might have a higher cell wall lytic enzyme than the original strain, which may account for the reduced biomass of the mutant. Thus, it is more beneficial to block spore formation at the asymmetric division stage than at the initial stage to increase the biomass of the cells as well as acetoin production.

### Construction of a robust chassis cell for AC production using lignocellulose hydrolysate

The *B. subtilis* occurs in autolysis in the middle and late stages of fermentation with increasing fermentation time, and this is particularly noticeable when lignocellulose is used as the fermentation substrate. The occurrence of cell lysis reduces the total biomass and decreases the fermentation yield [28]. Numerous factors mediate the lysis of *B. subtilis*, and we examined two more significant ones: intracellular cannibalism and peptidoglycan hydrolases. During fermentation, *B. subtilis* gradually produces spores as the nutrients decrease. The cells that prepare to form spores produce and release the toxic substances skf and sdp, which kill their non-spore-forming counterparts, and the nutrients released by the dead cells are used by the cells ready to form spores, a phenomenon we call cannibalism. The toxin production is mainly encoded by *skfA* and *sdpC* [29]. And the protein encoded by the *lytC* gene is considered the critical peptidoglycan hydrolase that mediates the autolysis of cells because the individual knockout of *lytC* effectively inhibits cell lysis [27, 30]. We took BSM-2 as the departure strain at this time and knocked out the relevant autolysis gene.

Analysis of the fermentation data showed that recombinant strain BSMA-1 with a double knockout of the *skfA* and *sdpC* genes did not significantly increase acetoin production, although it limited cell lysis to a certain extent. In contrast, knockout of the *lytC* gene in the recombinant strain BSMA-2 resulted in a significant increase in biomass by 18.3% compared to the original strain BS-1 in a medium with bagasse lignocellulose hydrolysate as the substrate, which ultimately led to a further increase in acetoin production, resulting in the titer of 33.7 g/L (Fig. 2A), an increase of 22.5% over the original strain (BS-1). This is because the knockout of the *lytC* gene increases the number of cells per unit volume in the fermentation broth and thus increases acetoin production, Wang et al. [29] also observed the same phenomenon when knocking out the gene *lytC* in *B. subtilis*. At the same time, we can see by transmission electron microscope that compared with the original strain BS-1, the recombinant strain BSMA-2 has no evident spores, but its cell morphology has changed significantly. The ends of the cells are darker in color, similar to the cellular areas of the forespores (Fig. 2C, D).

Compared to the original strain BS-1, the recombinant strain BSMA-2 optimized the intracellular carbon flux distribution, making the cells more conducive to acetoin production by fermentation. At the same time, the blocking of spores makes the recombinant strain easier to control during fermentation production and avoids pollution to the surrounding environment.

### Enhancing AC production by regulating intracellular redox homeostasis through Rex protein

When microorganisms use glycolysis to produce large amounts of pyruvate, they produce large amounts of NADH. Acetoin produced in microorganisms can use the NADH to reduce itself to 2,3-butanediol when too much NADH is made, resulting in much less efficient acetoin production [31, 32]. Previous studies have shown that *B. subtilis* contains a factor that responds to intracellular redox levels, called the redox-sensing global regulator Rex (encoded by the gene *ydiH*) [22]. Rex regulates intracellular redox homeostasis by sensing changes in the NADH/NAD^+^ ratio [33] and binds to the promoter regions of many genes to repress gene expression. This process can be inhibited by NADH and derepressed by excess NAD^+^. Most of these Rex-binding genes are involved in carbon and energy metabolism, including NADH oxidation, hydrogen production, ATP synthesis, and lactate and succinate formation [34, 35].

**Fig. 3:**
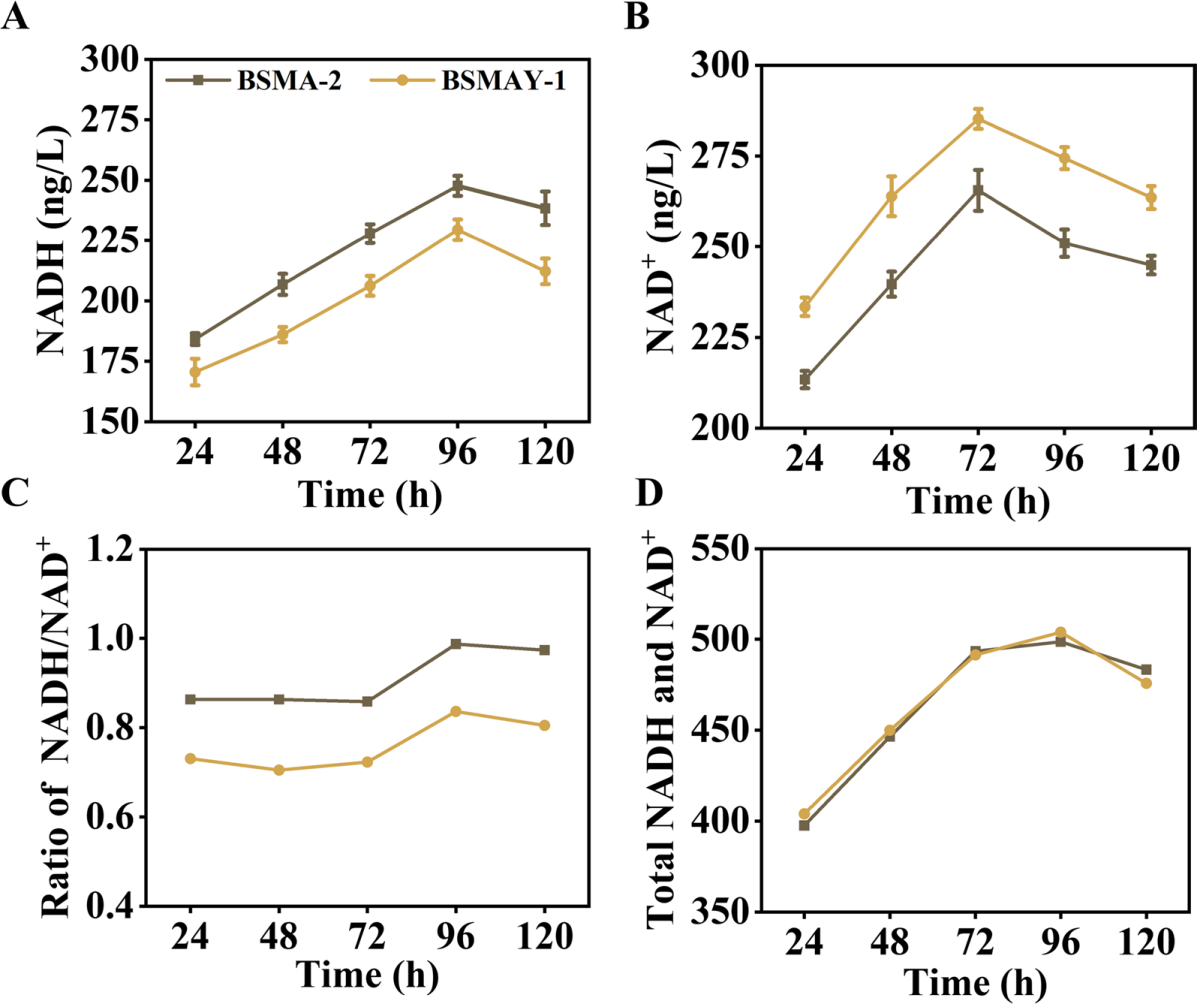
**A**–**D** Schematic representation of intracellular redox coenzyme-related levels after knockout of the *ydiH* gene. All assays were performed in triplicate, and the standard deviations of biological replicates are indicated by error bars

The construction of strain BSMAY-1 by knocking out the gene *ydiH* from strain BSMA-2. In order to reduce the inconvenience to experimental analysis caused by increased carbon source variables due to the complex composition of lignocellulosic hydrolysates in the experiment. In the section exploring metabolic engineering modification strategies to improve the conversion of substrates in recombinant bacteria, glucose was used as the sole carbon source. Fermentation data showed that knockout of the gene *ydiH* intensified the flow of carbon flux towards acetoin synthesis, which directly led to a reduction in the associated by-products of the NADH-dependent pathway. We followed up on the intracellular redox coenzymes, and the coenzymes data supported this phenomenon. The coenzyme data showed that knockout of the gene *ydiH* altered the intracellular concentrations of NADH and NAD^+^, resulting in a significantly lower NADH/NAD^+^ ratio than the starting strain, as shown in Fig. 3. This reduced ratio was beneficial for optimizing the intracellular carbon flow distribution and promoting acetoin production, which is consistent with the Li et al. results [36]. However, the growth rate of the bacterium was reduced at this time, probably due to the disruption of the intracellular homeostatic environment by knocking out *ydiH.* Although the knockout of Rex affected the growth rate of *B. subtilis* to a certain extent, it also affected the distribution of carbon flow and enhanced the production of acetoin and substrate conversion. The final acetoin titer of the BSMAY-1 strain with glucose as the sole fermentation substrate was 34.1 g/L, while the acetoin titer of the original strain BS-1 was 30.5 g/L under the same conditions.

### Combined blockage of competitive pathways increases AC production

Although the Rex protein adjusted the intracellular redox levels, the ability of the recombinant strain to produce AC was improved to some extent. However, there was still some accumulation of by-products at the end of fermentation, including 2,3-butanediol, lactic acid and succinic acid at concentrations of 6.2 g/L, 2.3 g/L and 1.4 g/L, respectively. In *B. subtilis*, the formation of 2,3-butanediol, lactic acid and succinic acid is mainly catalyzed by *bdhA*, *ldhA* and *mdh*, respectively [13]. In order to obtain a higher AC yield, we knocked out these genes combinatorially in strain BSMAY-1. The recombinant strain was then incubated at 37 °C in 250-ml shake flasks with glucose being the only carbon source, and fermentation was completed when glucose was almost wholly consumed.

The *bdhA* gene of BSMAY-1 was knocked out, and strain BSMAY-2 was obtained. Analysis of the fermentation data of strain BSMAY-2 showed a significant decrease in the yield of 2,3-butanediol. However, this component did not disappear completely, indicating other production pathways of 2,3-butanediol in *B. subtilis*. Subsequently, the gene *ldhA* was knocked out from strain BSMAY-2 obtaining strain BSMAY-3. And the knockout of the gene *ldhA* almost blocked the synthesis of lactate and further promoted the accumulation of acetoin. Interestingly, the knockout of the *mdh* gene obtaining strain BSMAY-4 increased acetoin production and increased the growth rate of the recombinant strain compared with the starting strain BSMAY-3, resulting in an increased acetoin production efficiency.

Through a series of metabolic engineering modifications, we finally obtained the recombinant strain BSMAY-4. After 84 h of shake-flask fermentation, the AC titer of strain BSMAY-4 increased to 40.1 g/L, as shown in Table 1, which was 40.98% higher than the original strain BS-1. The by-products 2,3-butanediol, lactic acid and succinic acid presented significant decreases, ending at 1.9 g/L, 0.2 g/L, and 0.5 g/L, respectively. This result is consistent with the modification of *Enterobacter cloacae* through metabolic engineering by Zhang et al. [6]. At the same time, the substrate utilization was also greatly improved, with a theoretical yield of 82.0%.

**Table 1:** Fermentation data for various recombinant strains

Strain	Acetoin (g/L)	2,3-Butanediol (g/L)	Lactic acid (g/L)	Succinic acid (g/L)	Residual glucose (g/L)	Equilibrium yield (mol/mol)
BSMAY-1	34.1 ± 0.3	6.2 ± 0.54	2.3 ± 0.1	1.4 ± 0.1	1.0 ± 0.2	0.70 ± 0.01
BSMAY-2	36.5 ± 0.8	1.7 ± 0.32	2.9 ± 0.2	1.4 ± 0.1	1.0 ± 0.3	0.75 ± 0.01
BSMAY-3	38.2 ± 0.5	2.0 ± 1.0	0.1 ± 0.1	1.6 ± 0.1	1.0 ± 0.2	0.78 ± 0.01
BSMAY-4	40.1 ± 0.2	1.9 ± 0.7	0.2 ± 0.1	0.5 ± 0.1	1.0 ± 0.3	0.82 ± 0.01

All assays are performed in triplicate and the standard deviation of biological replicates is expressed as a numerical error (note: the theoretical maximum of acetoin production per 100 g/L of glucose is 48.89 g/L, equilibrium yield = titer of acetoin/48.89 g/L)

### Enhancement of AC metabolic pathway carbon fluxes in mid-to-late fermentation

In the metabolic pathway of acetoin synthesis in *B. subtilis*, ALS and ALDC are responsible for catalyzing acetoin production from pyruvate and are encoded by the genes *alsS* and *alsD*, respectively. Previous studies in our laboratory revealed that the overexpression of *alsS* and *alsD* in *B. subtilis* using strong constitutive promoters inhibited the growth of the strain and was detrimental to acetoin production [23]. Therefore, we tried to use different auto-induced promoters, such as P_*ylb*_, P_*aprE*_, P_*cry3Aa*_, and P_*srfA*_, to overexpress *alsS* and *alsD* in the middle and late stages of fermentation to study their effects on acetoin synthesis.

**Fig. 4:**
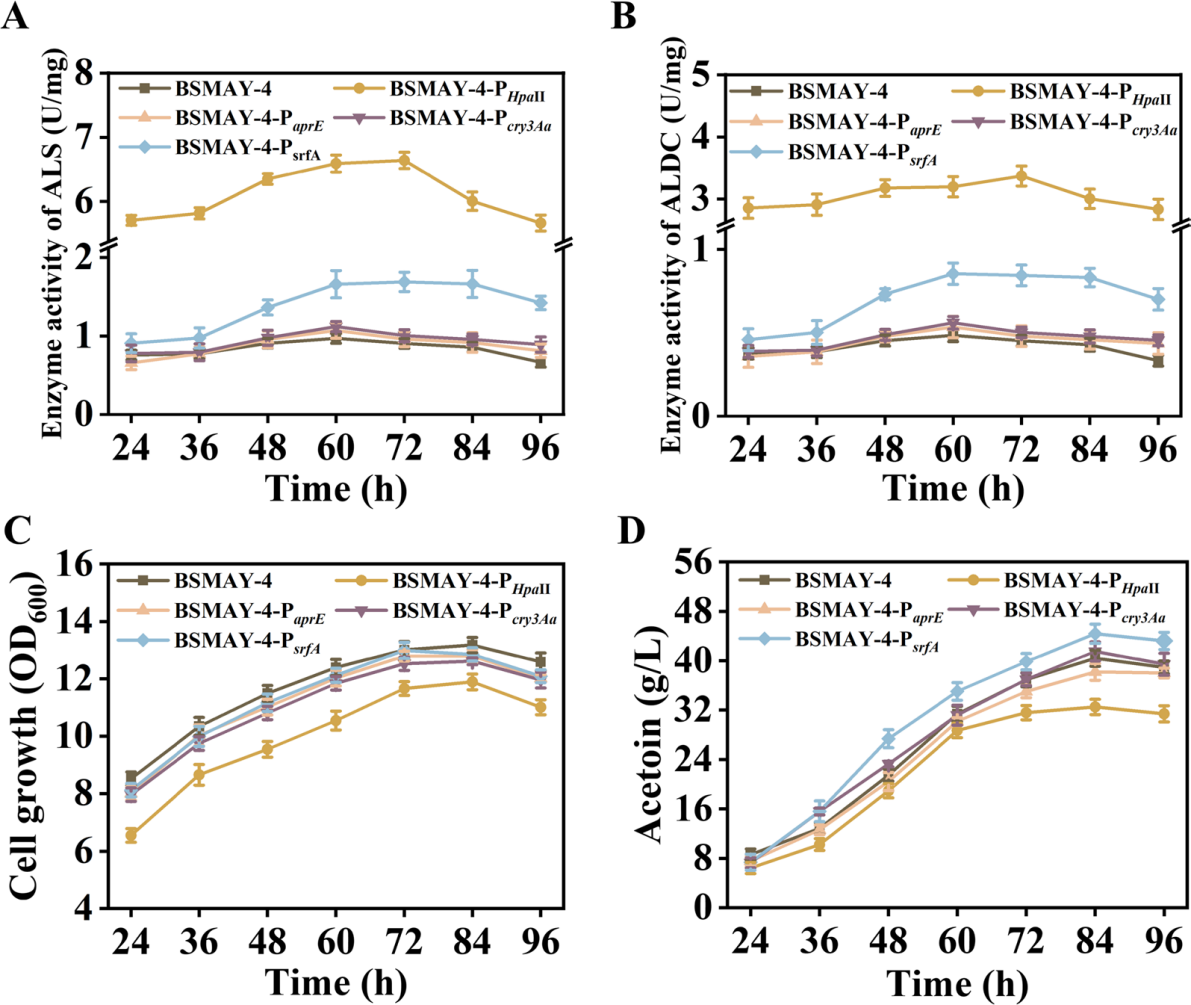
**A** and **B** Effects of various promoters on the activities of ALS (encoded by *alsS*) and ALDC (encoded by *alsD*), respectively. **C** Effects of different promoters on the growth of *B. subtilis* after overexpression of the *alsS* and *alsD* genes. **D** Effects of various promoters on acetoin production by *B. subtilis* after overexpression of the *alsS* and *alsD* genes. All assays were performed in triplicate, and the standard deviations of biological replicates are indicated by error bars

The recombinant expression plasmids pMA5-P_*Hpa*II_-*alsSD*, pMA5-P_*ylb*_-*alsSD*, pMA5-P_*aprE*_-*alsSD*, pMA5-P_*cry3Aa*_-*alsSD*, and pMA5-P_*srfA*_-*alsSD* were first transformed into strain BSMAY-4, and the enzyme activities of ALS and ALDC and the acetoin yield were measured periodically during fermentation. We found that when *alsS* and *alsD* were expressed using the constitutive strong promoter *Hpa*II in plasmid pMA5, the enzyme activities of ALS and ALDC in the strain were high throughout the fermentation process. In contrast, the growth of strain BSMAY-4-P_*ylb*_ was severely inhibited in the fermentation medium, and the maximum value of OD_600_ was only 10. Therefore, the enzyme activities and fermentation data were no longer measured for this strain. The data comparison revealed that the enzyme activities of ALS and ALDC in the recombinant strains BSMAY-4-P_*aprE*_ and BSMAY-4-P_*cry3Aa*_ were unchanged from the starting strain BSMAY-4 (Fig. 4A, B). The ALS and ALDC activities for the recombinant strain BSMAY-4-P_*srfA*_ were slightly higher than those for strain BSMAY-4 until 36 h. However, the activities of ALS and ALDC increased rapidly, reaching a maximum after about 60 h, which was almost twice those of the starting strain BSMAY-4. We subsequently performed shake-flask fermentation experiments for the strains BSMAY-4-P_*Hpa*II_, BSMAY-4-P_*aprE*_, BSMAY-4-P_*cry3Aa*_, BSMAY-4-P_*srfA*_, and BSMAY-4. The fermentation data indicated that when *alsS* and *alsD* were expressed in strain BSMAY-4 using the constitutive promoter *Hpa*II, the maximum biomass was reduced by 10.9% compared with the starting strain BSMAY-4 (Fig. 4C), while the acetoin titer reached only 32.5 g/L during the fermentation (Fig. 4D).

The lack of increase in acetoin yield may result from the synthesis of ALS and ALDC in large quantities during the early stage of fermentation competing with cell growth for substrates and coenzymes, thus limiting the cell growth and acetoin yield. When the population cell density-induced promoters P_*aprE*_ and P_*cry3Aa*_ were used to express *alsS* and *alsD*, bacterial growth was inhibited, with little overall change and only a limited increase in acetoin production. However, when *alsS* and *alsD* were expressed using the population cell density-induced promoter P_*srfA*_, which has weak activity in the early stage of fermentation, the *alsS* and *alsD* expression remained low and had little effect on bacterial growth. After 36 h of fermentation, the activation of P_*srfA*_ led to rapid increases in the ALS and ALDC activities, which promoted acetoin accumulation. The final acetoin titer for this strain reached 44.4 g/L, an increase of 55.8% compared to 28.5 g/L for the original strain BS-1, at which point the molar conversion reached 90.8% of the theoretical maximum yield.

The above results indicate that the enhanced expression of *alsS* and *alsD* in the mid-to-late fermentation stage using the population cell density-induced promoter solved the problem of inhibited growth when using strong promoters to express *alsS* and *alsD*. This significantly increased the acetoin yield while avoiding the use of inducers and reducing production costs. In addition, the improved expression of *alsS* and *alsD* enhanced acetoin anabolic flow and increased acetoin yield while lowering the production of other metabolic by-products to a certain extent.

### Fed-batch fermentation strain BSMAY-4-P_*srfA*_

**Fig. 5:**
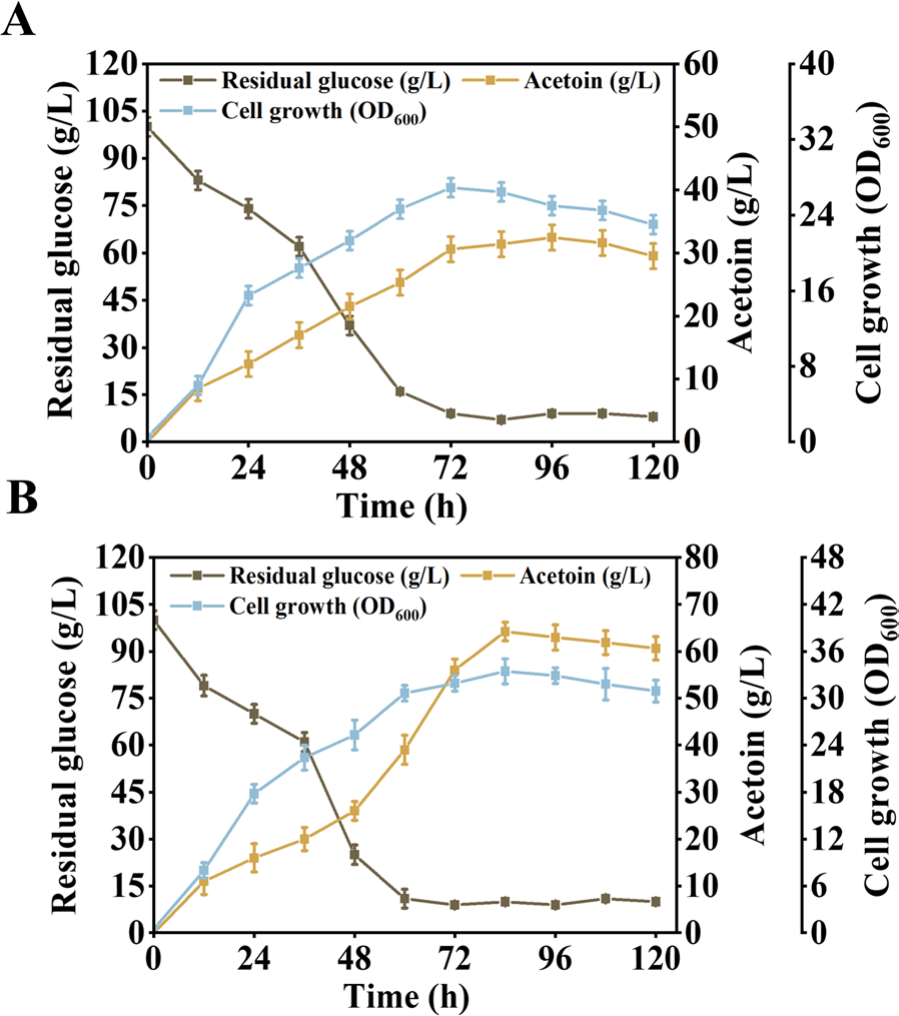
Acetoin production by fed-batch fermentation of **A** the original strain BS-1 and **B** the recombinant strain BSMAY-4-P_*srfA*_. All assays were performed in triplicate, and the standard deviations of biological replicates are indicated by error bars

The fermentation was carried out in batches using bagasse lignocellulosic hydrolysate as the carbon source for strain BSMAY-4-P_*srfA*_ and the original strain BS-1. As shown in Fig. 5A, the lignocellulosic hydrolysate was added to the fermentation broth to maintain the glucose concentration at no less than 10.0 g/L. After 84 h of fermentation, 64.3 g/L AC was obtained, and the yield of AC reached 89.5% of the maximum theoretical yield. In addition, the main by-products were 2,3-butanediol, lactic acid and succinic acid, which were obtained at concentrations of 3.4 g/L, 0.9 g/L and 0.7 g/L, respectively (Table 2).

**Table 2:** Fermentation data for the original strain BS-1 and the recombinant strain BSMAY-4-PsrfA

Strain	Acetoin (g/L)	2,3-Butanediol (g/L)	Lactic acid (g/L)	Succinic acid (g/L)	Equilibrium yield (mol/mol)
BS-1	32.5 ± 1.3	8.5 ± 0.5	4.6 ± 0.1	2.9 ± 0.1	0.55 ± 0.01
BSMAY-4-P_*srfA*_	64.3 ± 0.7	3.4 ± 0.7	0.9 ± 0.1	0.7 ± 0.1	0.90 ± 0.01

All assays are performed in triplicate and the standard deviation of biological replicates is expressed as a numerical error

The environmental and climate impacts of fossil fuels are of great concern. The fermentable sugars in lignocellulosic biomass are a potential source of fermentable sugars that can be converted into biochemical products and fuels. Therefore, the development of suitable fermentation strategies using cheap, non-food and renewable resources has great economic use.

## Conclusions

In this study, with sugarcane bagasse lignocellulose as the only fermentation substrate, the final acetoin titer for the recombinant strain BSMAY-4-P_*srfA*_ reached 64.3 g/L with the productivity of 0.765 g L^−1^ h^−1^ in a replenishment fractionated fermentation. As far as I know, this is the highest yield available for AC production by modified *B. subtilis* using bagasse lignocellulosic hydrolysate as a carbon source (Table 3). Key features of these strains include (1) knockouts of spore and autolysis genes that increase biomass and avoid waste of carbon sources; (2) Rex to regulate intracellular redox balance through redistribution of carbon flux; (3) blocking of competing pathways to increase acetoin yield; (4) enhancement of the acetoin pathway using the population cell density-induced promoter. In conclusion, compared to previous single-strategy modifications of model strains for acetoin production, this study uses a combination of optimized chassis cells and modular multi-strategy to redistribute intracellular carbon fluxes, providing a cost-effective and efficient approach for the industrial production of acetoin.

**Table 3:** Comparison of AC production by different *B. subtilis*

Strain	Method	Substrate	Concentration (g/L)	Yield (g/g)	Refs.
*B. subtilis* 168/pMA5-*zwf*	Overexpression of glucose-6-phosphate dehydrogenase in *B. subtilis* 168	Glucose	43.3	0.323	[45]
*B. subtilis* BSUW06	Deletion of *bdhA*, *acoA*, and *pta,* and overexpression of *alsSD* in *B. subtilis* 168	Glucose	19.8	0.396	[46]
BS-PAR	Moderating expression of the transcriptional regulator *AlsR* in *B. subtilis* 168	Glucose	41.5	0.346	[23]
*B. subtilis* IPE5-4-UD-4	By compound mutagenesis	Pretreated corncob	22.76	0.46	[47]
*B. subtilis* BMN	Inactivation of AR, moderate expression of *NOX*	Glucose	56.70	NA	[13]
*B. subtilis* BSK814A4	Deletion of *araR*, *bdhA*, and *acoA*; and insertion of a native *xyl* operon into the genome-reduced *B. subtilis* BSK814, which is derived from *B. subtilis* 168	Xylose	23.3	0.466	[4]
BSMAY-4-P_*srfA*_	Optimization of sump cell and intracellular coenzyme balance, combined with blocking of competing pathways and enhancement of *alsSD*	Pretreated bagasse Lignocellulose	64.26	0.437	This study

## Materials and methods

### Strains, plasmids, media, and materials

The primers used in the study and details of the strains are given in the Additional file 1. The gene-editing method of *B. subtilis* uses Cre/*lox*P site-specific gene operating system [37] and the competence of strains was prepared by reference [38]. The gene sequences of the promoters P_*ylb*_ [39], P_*aprE*_ [40], P_*cry3Aa*_ [41] and P_*srfA*_ [42] were obtained according to the reference and GenBank and gene synthesis. And primer designs were performed according to the gene sequences (see Additional file 1). The corresponding gene fragments were obtained by PCR reaction amplification. The plasmid pMA5 and the target gene fragment were double digested with endonucleases *EcoR* I and *EcoR* V (the enzymes *EcoR* V and *Kpn* I for the P_*ylb*_ fragment), and the purified gene fragment and pMA5 fragment were ligated to transform *E. coli* JM109. After the plasmid is successfully chemically transferred, extraction and sequencing are performed. Finally, recombinant vectors pMA5-P_*srfA*_-*alsSD*, pMA5-P_*aprE*_-*alsSD*, pMA5-P_*cry3Aa*_-*alsSD* and pMA5-P_*ylb*_-*alsSD* were obtained. The recombinant plasmid pMA5-P_*HpaII*_-*alsSD* was obtained by the same method. The constructed expression vector was transferred to *B. subtilis*, and finally a recombinant strain that could correctly express the target gene was obtained.

All recombinant plasmids and recombinant strains were constructed in lysogeny broth (LB) medium (10 g/L tryptone, 5 g/L yeast extract, and 10 g/L NaCl), with appropriate antibiotics (100 μg/mL ampicillin, 50 μg/mL kanamycin, or 30 μg/mL bleomycin) added if necessary.

The pretreated lignocellulosic hydrolysis product of bagasse contains 307.0 g/L glucose, 71.8 g/L xylose, small amounts of arabinose, mannose, and galactose, 6.2 g/L furfural, 3.3 g/L acetic acids, and small amounts of 5-hydroxymethylfurfural and formic acid (Shanghai Ting Pu Industrial Co kindly supplied them). The chemicals used in the experiments were purchased from Sangon Biotech (Shanghai, China). DNA gel purification kits, plasmid extraction kits, and bacterial genome rapid extraction kits were purchased from Generay Biotech (Shanghai, China). The 2 × Phanta Max Master Mix high-fidelity DNA polymerase for fragment amplification and colony Taq DNA polymerase for polymerase chain reaction were purchased from Vazyme Biotech (Nanjing, China). Restriction endonucleases were purchased from Takara Biotech (Beijing, China).

### Cultivation in shake flasks

First, single colonies were picked from LB solid medium plates and inoculated into 10 mL of LB liquid medium, then incubated in a shaker for 10 h at 37 °C. Next, 1 mL of this culture was seeded into a 50 mL LB liquid medium and incubated for 10 h at 37 °C. Finally, 3 mL of this culture was inoculated into 250-mL conical flasks containing 50 mL of acetoin fermentation medium (5 g/L yeast extract, 2 g/L urea, 15 g/L corn syrup, 100 g/L glucose solution, or lignocellulose sugarcane bagasse hydrolysate), which were fermented at 37 °C in a shaker at 180 rpm. The acetoin yield, OD_600_, and fermentation by-product yields were measured after sampling at regular intervals.

### Fed-batch fermentation conditions

The conditions for batch fermentation were as follows: fermenter volume of 5 L, inoculum volume of 6% of volume, fill volume of 2 L, aeration of 2 vvm, pH always controlled at 6.8, automatic addition of H_2_SO_4_ and NH_3_·H_2_O by using a computer-coupled peristaltic pump, fermentation temperature of 37 °C and dissolved oxygen concentration controlled at no less than 30% during the fermentation process. When the residual glucose in the fermentation broth dropped to approximately 10 g/L, the fermentation was split by adding lignocellulosic hydrolysate for replenishment.

Basal medium for batch fermentation (/L): 5 g yeast extract, 2 g urea, 15 g corn syrup, bagasse lignocellulosic hydrolysate at an initial glucose concentration of 100 g,2 g KH_2_PO_4_, 0.2 g CaCl_2_. Supplementary media (/L): 60 g corn syrup, bagasse lignocellulosic hydrolysate at an initial glucose concentration of 300 g.

### Analysis methods

The OD_600_ values and concentrations of glucose, acetoin, 2,3-butanediol, and each organic acid were determined as described in a previous study [13]. The ALS and ALDC enzyme activity assays were conducted according to a previous report [43]. Due to the CCR effect [44], *B. subtilis* preferentially uses glucose as the only carbon source, therefore, only the amount of glucose was calculated for the carbon source consumption in this experiment. The redox coenzymes NAD^+^ and NADH concentrations were measured using microbial NADH and NAD^+^ kits from Enzyme Link Biotechnology (Shanghai, China).

## Supplementary Information

**Additional file 1: Table S1.** The recombinant strains, recombinant plasmids, and primers were used in this work.

## Data Availability

All data generated or analyzed during this study are included in this published article and its additional files.
