# Supplementary material for: Acetoin production from lignocellulosic biomass hydrolysates with a modular metabolic engineering system in Bacillus subtilis
Source: Biotechnol Biofuels Bioprod. 2022 Aug 24;15:87. doi: 10.1186/s13068-022-02185-z (PMC9400278; doi:10.1186/s13068-022-02185-z)
Supplement: Supplementary file 1 — Additional file 1: Table S1. The recombinant strains, recombinant plasmids, and primers were used in this work. [file 13068_2022_2185_MOESM1_ESM.docx]

**Acetoin production from lignocellulosic biomass hydrolysates with a modular metabolic engineering system in *Bacillus subtilis***

Qiang Wang ^a,^ ^†^, Xian Zhang ^a, †,^ *, Kexin Ren ^a^, Rumeng Han ^a^, Ruiqi Lu ^a^, Teng Bao ^b^, Xuewei Pan ^a^, Taowei Yang ^a^, Meijuan Xu ^a^, Zhiming Rao ^a,^ *

*^a^ Key Laboratory of Industrial Biotechnology of Ministry of Education, School of Biotechnology, Jiangnan University, Wuxi, Jiangsu 214122, PR China*

*^b^ Department of Bioengineering, the University of Illinois at Urbana-Champaign, Urbana, Illinois, USA*

^†^ These authors contributed equally to this work.

* Corresponding author: Xian Zhang, E-mail: zx@jiangnan.edu.cn; Zhiming Rao, E-mail: raozhm@jiangnan.edu.cn

**Table S1.** The recombinant strains, recombinant plasmids, and primers were used in this work.

| Strain/plasmid | Genotype and description | Ref. |
| --- | --- | --- |
| ***Escherichia coli*** |  |  |
| JM109 |  | This study |
| JM109/pMA5-P*_Hpa_*_II_-*alsSD* | Amp^R^ | This study |
| JM109/pMA5-P*_srfA_*-*alsSD* | Amp^R^ | This study |
| JM109/pMA5-P*_aprE_*-*alsSD* | Amp^R^ | This study |
| JM109/pMA5-P*_cry3Aa_*-*alsSD* | Amp^R^ | This study |
| JM109/pMA5-P*_ylb_*-*alsSD* | Amp^R^ | This study |
| ***Bacillus subtilis*** |  |  |
| BS-1 | *B. subtilis* 168 | Our lab |
| BSM-1 | *B. subtilis* 168 knockouts of *spo0A* gene | This study |
| BSM-2 | *B. subtilis* 168 knockouts of *sigE* and *sigF* genes | This study |
| BSMA-1 | BSM-2 knockouts of *skfA* and *sdpC* gene | This study |
| BSMA-2 | BSM-2 knockouts of *lytC* gene | This study |
| BSMAY-1 | BSMA-2 knockouts of *ydiH* gene | This study |
| BSMAY-2 | BSMAY-1 knockouts of *bdhA* gene | This study |
| BSMAY-3 | BSMAY-2 knockouts of *ldhA* gene | This study |
| BSMAY-4 | BSMAY-3 knockouts of *mdh* gene | This study |
| BSMAY-4-P*_Hpa_*_II_ | BSMAY-4 containing plasmid pMA5-P*_Hpa_*_II_-*alsSD* | This study |
| BSMAY-4-P*_ylb_* | BSMAY-4 containing plasmid pMA5-P*_ylb_*-*alsSD* | This study |
| BSMAY-4-P*_aprE_* | BSMAY-4 containing plasmid pMA5-P*_aprE_*-*alsSD* | This study |
| BSMAY-4-P*_cry3Aa_* | BSMAY-4 containing plasmid pMA5-P*_cry3Aa_*-*alsSD* | This study |
| BSMAY-4-P*_srfA_* | BSMAY-4 containing plasmid pMA5-P*_srfA_*-*alsSD* | This study |
| **Plasmids** |  | This study |
| p7Z6 | pMD18-T containing gene lox71-zeo-lox66 |  |
| pDG148 | *E. coli* (Amp^R^), *B. subtilis* (Km^R^), a temperature-sensitive plasmid containing P*spac- cre* | Our lab |
| pMA5 | P*_Hpa_*_II_, *E. coli* (Amp^R^), *B. subtilis* (Km^R^) | Our lab |
| pMA5-P*_Hpa_*_II_-*alsSD* | Expression plasmid containing gene *alsSD* | This study |
| pMA5-P*_ylb_*-*alsSD* | Expression plasmid containing promoter P*_ylb_* and gene *alsSD* | This study |
| pMA5-P*_aprE_*-*alsSD* | Expression plasmid containing promoter P*_aprE_* and gene *alsSD* | This study |
| pMA5-P*_cry3Aa_*-*alsSD* | Expression plasmid containing promoter P*_cry3Aa_* and gene *alsSD* | This study |
| pMA5-P*_srfA_*-*alsSD* | Expression plasmid containing promoter P*_srfA_* and gene *alsSD* | This study |
| **Primers** | **Sequence (5ʹ–3ʹ) and enzyme cut site** | |
| Primers used for gene knockout | | |
| *spo0A*-L-F | ATAAACAGAAAATCAAAACGAAGCTGATCC | |
| *spo0A*-L-R | CCGAGCTCGAATTCGTAATCATGGTGTTTCTTCCTCCCCAAATGT | |
| *spo0A*-Z-F | ACCATGATTACGAATTCGAGCTCGG | |
| *spo0A*-Z-R | ACGTTGTAAAACGACGGCCAGTGCC | |
| *spo0A*-R-F | GGCACTGGCCGTCGTTTTACAACGTACATGAGCTTATTAAGTGGT | |
| *spo0A*-R-R | ATGAAAAACGATTAGCCTTCCCGCTCGCTA | |
| *sigE*-L-F | AGTCAGATGTGAAAATCTATTTAGATGTCA | |
| *sigE*-L-R | CCGAGCTCGAATTCGTAATCATGGTAAGTAACGGACATTTGCGAA | |
| *sigE*-Z-F | GATGTGAAAATCTATTTAGATGTCAACCATGATTACGAATTCGAG | |
| *sigE*-Z-R | TCAGGAAATCCCTGTAAAATCAAGGACGTTGTAAAACGACGGCCA | |
| *sigF*-R-F | GGCACTGGCCGTCGTTTTACAACGTTTTTGATAAGTGCTTTGTTA | |
| *sigF*-R-R | AGTACTCGCTGAAAGTCCTGTTGCTGCA | |
| *sigF*-L-F | CCGACGAACAAACCTGCCAGAAGCCCGACC | |
| *sigF*-L-R | CCGAGCTCGAATTCGTAATCATGGTATTTATGGTCTTTTCGAGCG | |
| *skfA*-L-F | TGCACTGAAGCTGAATGAGATTTCTTAAG | |
| *skfA*-L-R | AAGTAAACCTCCTCTCAATTTTTGCATAGAGT | |
| *skfA*-Z-F | ACTCTATGCAAAAATTGAGAGGAGGTTTACTTgaccatgattacgaattcgagct | |
| *skfA*-Z-R | CAAATACGCTCAACTCCCTATTCTCAAATGtgtaaaacgacggccagtgcca | |
| *skfA*-R-F | CATTTGAGAATAGGGAGTTGAGCGTATTTG | |
| *skfA*-R-R | GGAATGTTTGCTTCTGATAATTTTTTAATGGTATTCATAC | |
| *sdpC*-L-F | ATTTTGAGATTATAAGATACTTAATGATTT | |
| *sdpC*-L-R | ATTATTATACCTCCATTAAGTTATTTCTCC | |
| *sdpC*-Z-F | GGAGAAATAACTTAATGGAGGTATAATAATgaccatgattacgaattcgagctcggtacc | |
| *sdpC*-Z-R | GCAATCCGCAAGACACTCAATTATAATGGAtgtaaaacgacggccagtgccaagctt | |
| *sdpC*-R-F | TCCATTATAATTGAGTGTCTTGCGGATTGC | |
| *sdpC*-R-R | TTTTAAAGCAAGCGGAAGTCATAAGTGACC | |
| *lytC*-L-F | TAAAGTGAAAGTCAGATTGCGAATGTAATA | |
| *lytC*-L-R | TCGAAAGAGACAAATCTAATCACAGATTT | |
| *lytC*-Z-F | AAATCTGTGATTAGATTTGTCTCTTTCGAgaccatgattacgaattcgagctcggt | |
| *lytC*-Z-R | GGATATAACGCCATTTAAGGAGGAAATCAAtgtaaaacgacggccagtgccaagcttgc | |
| *lytC*-R-F | TTGATTTCCTCCTTAAATGGCGTTATATCC | |
| *lytC*-R-R | AAAACAGGAACTACTGTACCTGATACA | |
| *ydiH*-L-F | catacgaaagctggcctgcgataattttaaggagcgtggattttc | |
| *ydiH*-L-R | aatcatggtcttttggtcctccaaattatactcggatagttctcttttaaagtcaccg | |
| *ydiH*-Z-F | ccgagtataatttggaggaccaaaagaccatgattacgaattcgagctcggtacccgg | |
| *ydiH*-Z-R | gcatatttgggctcctcctttcccttgtaaaacgacggccagtgccaagct | |
| *ydiH*-R-F | ggcactggccgtcgttttacaagggaaaggaggagcccaaatatgccgat | |
| *ydiH*-R-R | agcgtaaaatacgcatactttctgattttcgccaagaacatcggt | |
| *bdhA*-L-F | CTTTCGGTATTGACTGAAACATCAGCAGCC | |
| *bdhA*-L-R | CCGAGCTCGAATTCGTAATCATGGTACCGGTCAATATGAACTGTT | |
| *bdhA*-Z-F | CTCGGAACAGTTCATATTGACCGGTACCATGATTACGAATTCGAG | |
| *bdhA*-Z-R | GTGCCGTGATTGGATAATTTAAAAAACGTTGTAAAACGACGGCCA | |
| *bdhA*-R-F | GGCACTGGCCGTCGTTTTACAACGTTTTTTAAATTATCCAATCAC | |
| *bdhA*-R-R | TTTTTAATGCGATCCTTGGAACAACAATGGG | |
| *ldhA*-L-F | TGGCTGGACAGCCTGAGGAACTCTCGAACC | |
| *ldhA*-L-R | CCGAGCTCGAATTCGTAATCATGGTTGTTTGTGAAGTATTTCACA | |
| *ldhA*-Z-F | ATAAATGTGAAATACTTCACAAACAACCATGATTACGAATTCGAG | |
| *ldhA*-Z-R | TCCCACATTGACAATCAGCCCTTTAACGTTGTAAAACGACGGCCA | |
| *ldhA*-R-F | GGCACTGGCCGTCGTTTTACAACGTTAAAGGGCTGATTGTCAATG | |
| *ldhA*-R-R | CAGCCCGCCTTCTTGGAAAAGCGCTTTGAAG | |
| *mdh*-L-F | ATCTCCGGCAAAATCATAATTCCAGAC | |
| *mdh*-L-R | TAAAAAGAGAGAAAGGCTTGCTTAATACAGC | |
| *mdh*-Z-F | GCTGTATTAAGCAAGCCTTTCTCTCTTTTTAGACCATGATTACGAATTCGAGCTCG | |
| *mdh*-Z-R | CCTAAAACTAGCCATAAAGGAGAAGAGAGACTGTAAAACGACGGCCAGTGCC | |
| *mdh*-R-F | GTCTCTCTTCTCCTTTATGGCTAGTTTTAGG | |
| *mdh*-R-R | CAGCTTCCTTCAAAATGAGTTAAACGTCAA | |
| *alsSD*1-F | CCG***CTCGAG***TTGACAAAAG CAACAAAAG(*Xho*I) | |
| *alsSD*1-R | ATAAGAAT***GCGGCCGC***TTATTCAGGG CTTCCTTCA(*Not*I) | |
| *alsSD*2-F | ACCG***GGATCC***TTGACAAAAGCAACAAAAGAAC(*Bam*HI) | |
| *alsSD*2-R | ACCG***ACGCGT***TTATTCAGGGCTTCCTT(*Mlu*I) | |
| P*_ylb_*-L | ACCG***GATATC***CATCGTCGAACGCGCTCCA(*Eco*RV) | |
| P*_ylb_*-R | CGG***GGTACC***GCGGCCGCACTCGAGACGTTCTACCTTTGTCAAA (*Kpn*I) | |
| P*_aprE_*-L | CCG***GAATTC***CTTATTTCTTCCTCCCTCTC(*Eco*RI) | |
| P*_aprE_*-R | GCC***GATATC***GCGGCCGCACTCGAGCACTCTTTACCCTCT (*Eco*RV) | |
| P*_cry3Aa_*-L | CCG***GAATTC***GTGCTTTTTTTGTTGAC(*Eco*RI) | |
| P*_cry3Aa_*-R | GCC***GATATC***GCGGCCGCACTCGAGTTTTCTTCCTCCCTTT (*Eco*RV) | |
| P*_srfA_*-L | CCG***GAATTC***ATCGACAAAAATGTCATG(*Eco*RI) | |
| P*_srfA_*-R | GCC***GATATC***GCGGCCGCACTCGAGATTGTCATACCTCCCCTAAT(*Eco*RV) | |
